# Design and Fabrication of a MEMS Flow Sensor and Its Application in Precise Liquid Dispensing

**DOI:** 10.3390/s90604138

**Published:** 2009-06-02

**Authors:** Yaxin Liu, Liguo Chen, Lining Sun

**Affiliations:** State Key Laboratory of Robotics and System, Harbin Institute of Technology, Harbin, China; E-Mails: clg@hit.edu.cn (L.C.); lnsun@hit.edu.cn (L.S.)

**Keywords:** flow sensor, MEMS, liquid dispensing

## Abstract

A high speed MEMS flow sensor to enhance the reliability and accuracy of a liquid dispensing system is proposed. Benefitting from the sensor information feedback, the system can self-adjust the open time of the solenoid valve to accurately dispense desired volumes of reagent without any pre-calibration. First, an integrated high-speed liquid flow sensor based on the measurement of the pressure difference across a flow channel is presented. Dimensions of the micro-flow channel and two pressure sensors have been appropriately designed to meet the static and dynamic requirements of the liquid dispensing system. Experiments results show that the full scale (FS) flow measurement ranges up to 80 μL/s, with a nonlinearity better than 0.51% FS. Secondly, a novel closed-loop control strategy is proposed to calculate the valve open time in each dispensing cycle, which makes the system immune to liquid viscosity, pressure fluctuation, and other sources of error. Finally, dispensing results show that the system can achieve better dispensing performance, and the coefficient of variance (CV) for liquid dispensing is below 3% at 1 μL and below 4% at 100 nL.

## 1. Introduction

During the past decades, transfers of liquid volumes in the submicroliter range have become an important feature of liquid handling robotic instruments for protein crystallization, drug discovery, and medical diagnostics. In order to dispense smaller volumes than that be dispensed by hand with more accuracy, higher speed, and better reproducibility, many automated liquid dispensing technologies have been developed in academic and commercial applications [1-2]. In the early 1990s, the contact dispensing technologies capable of delivering fluid volumes in the submicroliter range appeared. Initially these systems featured piston displacement mechanisms, but displacement techniques do not provide enough energy to break the surface tension of the last droplet, so a dragging action, touch off (against either the solid surface of a vessel or a liquid surface) is employed. Then inkjet dispensing technology was introduced, along with syringe-driven positive displacement technology [3-4]. Inkjet technology alleviates some problems of contact dispensing by forcing the sample through a small opening and projecting it onto the slide surface in a contactless manner. Inkjet dispensers include two main types: piezoelectric and solenoid based systems. Piezoelectric-based systems (Packard Instruments, among others) use piezoelectric crystals coupled to a glass capillary tube [5]. Solenoid-based systems (Cartesian Technology, Innovadyn Technology, among others) use pressure to compress the fluid against a valve [6]. In addition, some novel liquid handling technologies that feature electrical conductivity gradient, thermally actuated, and focused acoustics mechanisms have also been developed [7-14]. However, a number of critical aspects on low-volume liquid handling remain unresolved.

In some application conditions, for example, protein crystallization, many reagents with different viscosities need to be dispensed during one screening experiment, but most of the commercial automated liquid dispensing systems cannot adjust system parameters automatically, and a dispensing device operating will dispense either more liquid with lower viscosity or less of a higher viscosity liquid, so dispensing volume errors are introduced when liquids of different viscosities are handled simultaneously.

In order to solve this problem, most commercial automated liquid dispensing systems ensure precision by experimental calibration when liquid viscosities change, which is time consuming and less flexible. Recently, a variety of sensors have been used in high-throughput liquid dispensing systems. In 2000, a Bluebird™ dispenser was reported to improve accuracy by using a pressure feed-back loop [15]. In 2005, Carsten Haber tried to integrate a MEMS flow sensor in the Seyonic system, and first proposed a residual volume compensation strategy to constantly monitor and correct the dispensing process for accurate fluid delivery during dispensing cycles [16]. Integrating the sensors make it possible to dispense the desired volumes of liquids with different viscosities accurately by closed-loop control.

**Figure 1.**
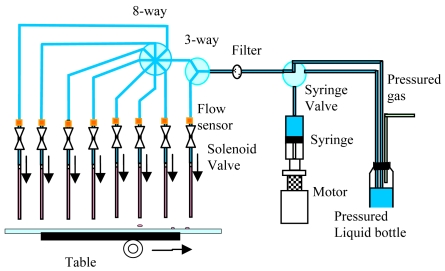
The schematic of the non-contact adaptive precise liquid dispensing system.

In this paper, an adaptive precise liquid dispensing system with a more intelligent control approach was developed. It consists of a syringe pump, syringe valve, pressurized reagent bottle, pressure regulator, microsolenoid valves, and sensors, etc, as shown in Figure 1. A MEMS flow sensor was designed, fabricated, and integrated in the liquid dispensing system. Besides, an advanced compound fuzzy control strategy was introduced to control the valve open time in each dispensing cycle. With feedback information from the flow sensor, the dispensing system could self-adjust the open time of the solenoid valve automatically so as to dispense the desired volumes of reagents over a large range of viscosities, as well as detect air bubbles or nozzle clogs in real time. First, the design, fabrication, and calibration of the key component in dispensing system (the flow sensor) are introduced in detail. Then, the compound fuzzy control strategy is expounded. Finally, the experimental results are given to show the precision of this liquid dispensing system.

## 2. The MEMS flow sensor

### 2.1. Design and Fabrications

**Figure 2.**
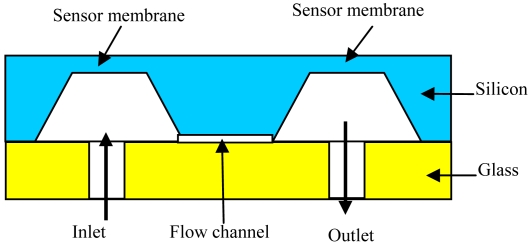
The layout of the sensor chip.

In the proposed liquid dispensing instrument, an integrated high-speed liquid flow sensor based on the measurement of pressure difference across a flow restriction is presented. It provides closed-loop control for accurately dispensing liquids over a large range of viscosities, as well as detecting air bubbles or nozzle clogs in real time. The functional layout of the sensor chip is shown in Figure 2.

The sensor chip consists of two piezo-resistive sensor dies and a micro-machined channel. By use of anodic bonding process, the glass wafer is mounted on the silicon wafer. The pressure drop induced by liquid flow across the micro-machined channel at low Reynolds numbers is expressed as in (1) [17]:

(1) $$\Delta P=Q_{v}\times \frac{C\mu L}{2A{D_{h}}^{2}}$$

where Δ*P* is the pressure drop (Pa), *Q_v_* is the volumetric flow rate (m^3^/s), *C* is a dimensionless friction factor [1], *μ* is fluid dynamic viscosity (Pa.s), *L* is the channel length (m), *A* is the channel cross section (m^2^), and *D_h_* is the equivalent hydraulic diameter (m).

**Figure 3.**
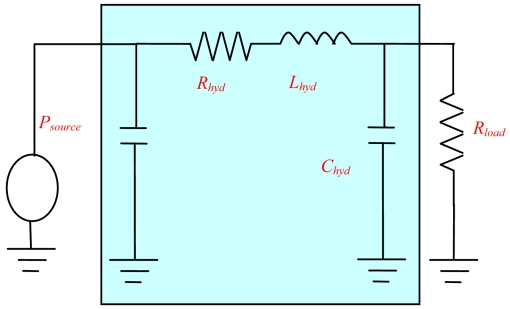
Electric analogy of the flow sensor.

Based on (1), the flow rate can be obtained from the pressure drop. In the liquid dispensing system, the sensor is positioned between the pressure source and the solenoid valve, where only the system fluid (de-ionized water) flows through. Thus the sensor's sensitivity is determined by the physical dimensions of the flow channel. In addition, the flow sensor is operated with high speed fluid in the dispensing system, so it is important to know its dynamic behavior for predicting the time dependent signal from the flow and pressure. A lumped electric element analogy of the flow sensor was used to estimate the working range, as shown in Figure 3.

From Figure 3 the transfer function and resonance frequency *f_hyd_* of the LRC circuit can be deduced as in (2) and (3). Then it can be concluded that it is possible to increase *f_hyd_* by reducing *C_hyd_* and *L_hyd_* so as to obtain a higher dynamic range. Due to the square membrane deflection under a pressure load, liquid can be accumulated. The hydraulic capacity of the sensor is expressed in (4). The inertance of the sensor caused by the acceleration of liquid mass can be defined as in (5):

(2) $$G_{\text{hyd}}=\frac{1}{1+j\omega R_{\text{hyd}}C_{\text{hyd}}-\omega^{2}L_{\text{hyd}}C_{\text{hyd}}}$$

(3) $$f_{\text{hyd}}=\frac{1}{2\pi \sqrt{L_{\text{hyd}}C_{\text{hyd}}}}$$

(4) $$C_{\text{hyd}}=0.28\frac{{\left(a/2\right)}^{6}}{Eh^{3}}\left(1-v^{2}\right)$$

(5) $$L_{\text{hyd}}=\frac{\rho l}{A}$$

where *L* is the channel length, *A* is the channel cross section, *E* is the modulus of elasticity, *v* is the Poisson constant, *ρ* is the density, *a* is the membrane width, *h* is the membrane thickness. It can be seen that the dynamic behavior is influenced by the stiffness of the membrane and the dimension of the channel.

**Figure 4.**
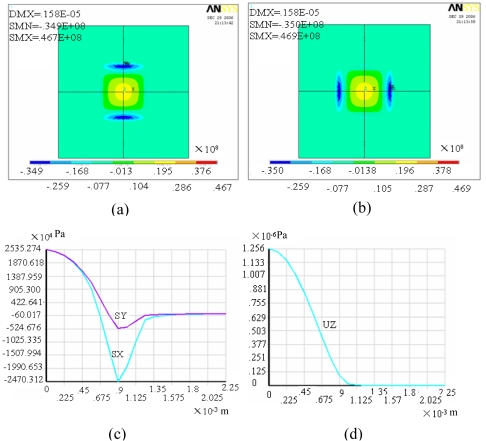
Simulation shows the stress and strains for single membrane under 15 psi pressure.

Based on the principles above, the sensor prototype was designed. It consists of two square silicon membranes with dimensions of 50 μm thick × 2,000 μm wide × 2,000 μm long, and the *C_hyd_* value is around 1.60×10^-17^m^5^/N. Simulation of a single membrane by ANSYS (general purpose finite element analysis software) is shown in Figures 4(a) and 4(b), and the stresses and strains on X-axis path are shown in Figures 4(c) and 4(d). From the results we can see that the stress at maximum system pressure (15 psi) is 2.5×10^7^ Pa, which is less than the limit value 80 MPa, and the maximum deflection is 1.3 μm, which is far smaller than the membrane thickness of 50 μm. Therefore the membranes stay in elastic deformation stage. In the liquid dispensing system, the required liquid flow rate is about several 10 μL/s. So the channel is designed as 2,005 μm long and 30 μm deep. For a 2,000 μm wide channel, the resistance to water is 3.36×10^11^ Ns/m^5^ with an inertia of 3.2×10^7^ kg/m^4^. Besides, the resonance frequency is 7,019 Hz, and then the sensor can work well with a fluid frequency up to 1 kHz.

**Figure 5.**
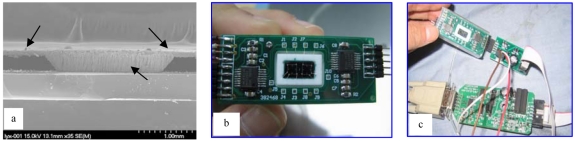
(a)The SEM picture of the sensor chip cross-section. (b) The photo of the sensor packaging. (c) The communication module for calibration.

The sensor fabrication consists of an industrial piezo-resistive process with the addition of an extra backside anisotropic etch step to integrate the flow channel. Firstly, piezoresistances are placed in a Wheatstone bridge by ion implantation. Then the membranes and the channel are anisotropic etched by use of double lever mask technique. After this, the contact hole was formed on the top side by buffer by HF acid etching, and then aluminum is sputtered and patterned. Finally, the glass wafer with 1.8 mm holes is mounted on the silicon wafer by an anodic bonding process. The holes on the glass are created by using an ultrasonic machining process. A SEM picture of the sensor chip cross-section is shown in Figure 5(a). The finished sensor chip is mounted on a ceramic substrate where all the compensating electrical circuits and fluidic connections are placed, and Figure 5(b) shows the packaging configuration. Outputs of sensor chip are magnified by two signal-conditioner Ics (MAX1452 from Maxim Company). Before being used, the sensor should be connected to the communication module for calibration as shown in Figure 5(c). In the next part, the calibration process will be described in detail.

### 2.2. Calibrations

**Figure 6.**
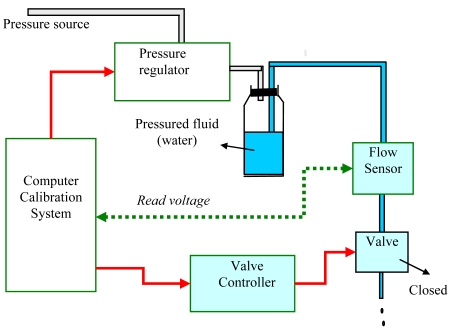
Experimental set up for pressure-voltage calibration.

The pressure-voltage calibration system is used to supply a controlled constant pressure. It mainly consists of a computer, pressure regulator, pressurized fluid bottle, flow sensor, solenoid valve, and valve controller, and the experimental system configuration is shown in Figure 6. In this system, the computer sends a command to make the pressure regulator output pressed air with settled pressure. Then the compressed air fills a 2 L solvent bottle, and pushes the water that then flows through the flow sensor and valve.

**Figure 7.**
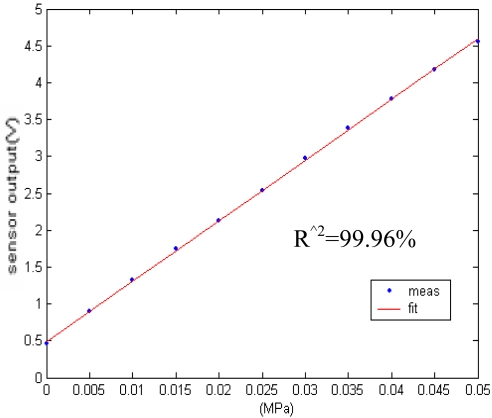
Measured output voltages for different pressures, the ‘goodness of fit’ statistics is 99.96%.

During calibration, one must ensure that the electrical circuit module of the sensor is connected to the communication module and the communication module is connected to the computer. Using the corresponding calibration software of the MAX1452 unit, the sensor can be calibrated via communication to the computer. For the operational details the reader is referred to the MAX1452 user manual. After that, the communication module is removed, leaving the electrical circuit module to work alone. At this time, the MAX1452 switches to analog mode, and the voltage output of pressure measuring element in the sensor has been calibrated to the desired value. The calibrated output signal under different system pressures is shown in Figure 7. Based on the deviation between calibration curve and the fitting curve, the nonlinearity of pressure sensor can be calculated as 0.29%.

**Figure 8.**
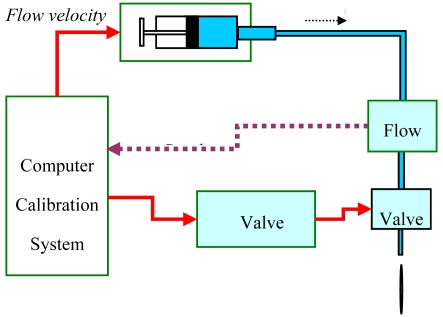
The schematic of experimental set up for flow-voltage calibration.

The flow-voltage calibration system mainly consists of a computer, syringe pump, flow sensor, solenoid valve, and valve controller, as shown in Figure 8. The syringe pump can supply 40 flow rates ranging from 0.4167 μL/s up to 300 μL/s. For each flow condition, the corresponding average output voltage was recorded.

**Figure 9.**
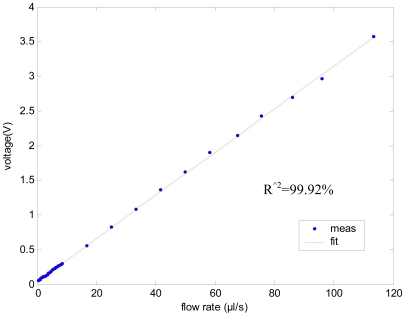
Sensor voltage output for different flow rate, the ‘goodness of fit’ statistics is 99.92%.

The output voltage differences for different flow rate are shown in Figure 9, where the sensitivity of the calibrated sensor is 31 mV/(μL/s). Based on the deviation between calibration curve and the fitting curve, the nonlinearity of flow sensor can be calculated as 0.51%.

## 3. Feedback control Loop

**Figure 10.**
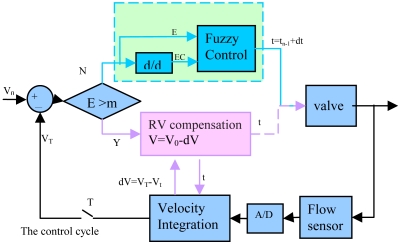
The schematic of the intelligent compound control strategy.

To adjust the open time of the solenoid valve to automatically sample viscosity with sensor feedback, a more intelligent compound control strategy is proposed, as shown in Figure 10. This strategy enables the liquid dispensing system to dispense smaller volumes of reagent, and be immune to sample viscosity, pressure fluctuation, and other disturbances. In each dispensing cycle *T_n_*, as the valve is triggered, the system begins to integrate the feedback flow velocity. Then both the actual dispensed volume *V_T_* of each cycle and the dispensed volume *V_t_* during valve open time can be calculated via real-time flow pulse integration. In the next dispensing cycle *T_n+1_*, if the volume error between *V_0_* and *V_T_*, namely *E*, is larger than critical value *m*, then the new valve open time will be set via a Residual Volume (RV) Compensation strategy; otherwise it will be set by a fuzzy control strategy.

The RV Compensation strategy mentioned above mainly considers the effect of residual volume, which is similar to that proposed by Haber. For example, the desired volume is *V_0_*, and the valve will be open for an interval until the *V_0_* is dispensed. However, when the valve is closed, the flow rate will not be reset to zero immediately, which causes a redundant volume d*V* to be dispensed and that the overall dispensed volume is *V_0_*+d*V*. Hence, in the next dispensing cycle, the residual volume will be taken into account, and the desired volume is considered as *V_0_*-d*V*. At the dispense trigger, the system begins to integrate the flow pulse, and closes the valve at the exact moment when the new set desired volume has been dispensed, thus the requested volume can be dispensed accurately.

**Figure 11.**
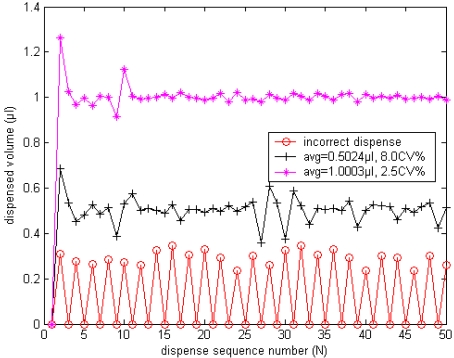
The dispensed volume in each cycle with only RV Compensation strategy.

For the RV Compensation strategy, it can be seen that the minimum controlled dispensed volume is determined by the residual volume. When the residual volume exceeds the requested volume, the system is not capable to deliver the correct volume any longer. Especially in the non-contact dispensing process, flow rates are normally higher than 20 μL/s, which results in that approximately 200–250 nL of residual volume is dispensed after valve is closed, so it is difficult to dispense correct volumes smaller than 200 nL only using RV Compensation strategy in this system. Figure 11 shows the actual dispensed volumes for initial 50 cycles with the conventional RV compensation strategy only. It can be seen that the precision deteriorates as the dispensed volume is reduced. When the desired volume is reduced to 0.1 μL, the system cannot work correctly with only the conventional RV compensation strategy.

Therefore a fuzzy controller is introduced to dispense smaller desired volumes. If the volume difference is smaller than the critical value *m*, the valve open time will be determined by the fuzzy controller. The input variables of the fuzzy controller are volume error (*E*) and volume error change rate (*EC*), and the output variable is the change of valve open time (*U*). Then the analytic control rule in fuzzy control can be summed up as (6):

(6) U=−<(αE+(1−α)EC)>

where *α* is the rectification factor that can be designed as (7):

(7) α=|E|/|E|+|EC|

In each dispensing cycle, the change of valve open time (*dt*) for the next cycle can be calculated. Thus, in the next dispensing cycle, the open time of the solenoid valve (*t*) is expressed as (8):

(8) $$t=t_{n-1}+dt$$

**Figure 12.**
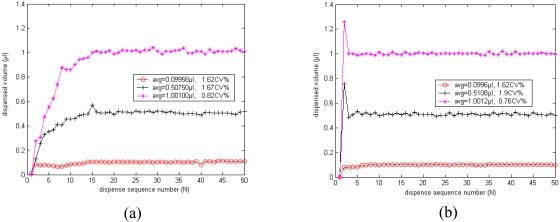
(a) The dispensed volume in each cycle with only fuzzy control strategy, (b) The dispensed volume in each cycle with intelligent compound control strategy.

For fuzzy control, the minimum controllable dispensed volume depends on the valve response time rather than the residual volume, and the system will not run away until the calculated valve open time is smaller than the valve response time. So the system can dispense smaller volume with fuzzy control strategy. Figure 12(a) shows the actual dispensed volumes in initial 50 cycles with fuzzy control strategy only. It can be seen that the precision does not get worse with reduction of dispensing volume, and the system can also dispense exact volume despite the small desired volume of 0.1 μL. However, the system still has shortcomings; it must pre-dispense several times to achieve the desired volume.

Therefore an intelligent compound control strategy which combines residual volume compensation method and fuzzy control strategy is proposed, as shown in Figure 10. If the volume error *E* is larger than the critical value *m*, the new valve opening time will be set via the RV Compensation strategy; otherwise it will be set by a fuzzy control strategy. Thus the system can quickly dispense volumes that approach the desired volume in the first dispensing cycle. Figure 12(b) shows the actual dispensed volumes in the initial 50 cycles with this intelligent compound control strategy. It can be seen that by use of the intelligent compound control strategy, the system can not only dispense smaller volumes, but also dispense volumes close to the desired volume in the first cycle.

The intelligent compound fuzzy control strategy also has some other merits. In a high throughput liquid dispensing process, the viscous reagent in the pipe reduces gradually, which makes the identical viscosity decrease gently; and the system pressure may also fluctuate during the dispensing process, which would affect the dispensing precision. Fortunately, the intelligent compound fuzzy control strategy is a model free design approach and it makes the liquid dispensing system immune to sample viscosity, pressure fluctuation, and some other disturbances. Compared to the use of only RV Compensation strategy, the system using the intelligent compound control strategy has a better precision.

**Figure 13.**
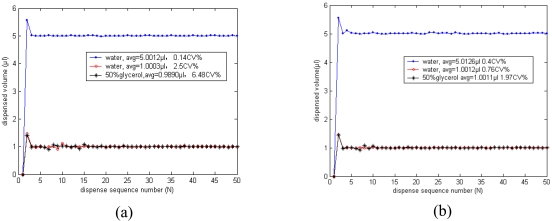
Dispensed volume for each cycle with different control strategies, desired volume and reagent.

Finally, water and 50% glycerol were dispensed 50 times with different control strategies, and the dispensed volume for each cycle was recorded. Figure 13(a) shows the actual dispensed volumes in the initial 50 cycles with the conventional RV compensation strategy only. The reproducibility becomes 2.54CV% when the dispensed volume reduces to 1 μL, and it is 6.48CV% when dispensing more viscous 50% glycerol. Oppositely, by use of the novel compound intelligent control strategy, the system dispensing reproducibility is still below 1.97CV%, as shown in Figure 13(b).

From the comparison experiments above, we can see that the novel intelligent control strategy combines the advantages of residual volume compensation method and the fuzzy logic control method. By use of this strategy, the system can find appropriate valve open time through only one pre-dispensing cycle without manual calibration, and constantly monitor as well as adjust the dispense process to sample viscosity, pressure fluctuation, and some other disturbances. Moreover, with the real-time information from the flow sensor, air bubbles or nozzle clogs can be diagnosed; if nozzle clogs happen, a higher pressure can be detected by the sensor, and the system will report it at once. And the liquid dispensing performance as well as each dispensing channel status can be instantly monitored and reported so as to ensure dispensing reliability.

## 4. Dispensing experiments and results

Dispensing experiments were carried out with different volumes and reagents in 96-well plate. The experiment reagents were water, 10% glycerol, 20% glycerol, 30% glycerol, 40% glycerol, and 50% glycerol. For each reagent, the dispensing precision at 0.1, 0.5, 1 and 5 μL were tested and recorded, as shown in Table 1.

The precision of the adaptive precise liquid dispensing system was evaluated by the coefficient of variance (CV %). And Table 1 shows that coefficient of variance is below 3% at 1 μL and below 4% at 100 nL.

## 5. Conclusions

A flow sensor based on measurement of pressure drop across a micro-channel has been developed to enhance the reliability and accuracy of liquid dispensing system. In this paper, the design, fabrication, packaging, and characterization of the differential pressure flow sensor were presented. Formulas were derived to predict the static and dynamic behavior of sensor, and dimensions of the two silicon cups and micro-channel were designed properly for the liquid dispensing system.

Calibration results show that it allows measurement of flow rates up to 80 μL/s and the FS pressure drop is less than 5 psi. The sensitivity of the calibrated sensor is 31 mV/(μL/s) and the nonlinearity is better than 0.51 %FS. The advanced intelligent compound fuzzy control strategy is introduced in detail. Dispensing results show that the system can achieve better dispensing performance by use of a compound intelligent fuzzy control strategy. Integration of the custom high-speed MEMS flow sensor and use of the compound intelligent control strategy make it possible to self-adjust the open time of the solenoid valve for dispensing desired volume reagents accurately with a large range of viscosities. Furthermore, it could constantly monitor and correct the dispensing process to make this system immune to sample viscosity, pressure fluctuation, and some other disturbances. Finally, the dispensing experiment results show that the coefficient of variance (CV) for liquid dispensing is below 3% at 1 μL and below 4% at 100 nL.

## Figures and Tables

**Table 1.** Reproducibility of dispensing.

Reagent	Dispense Reproducibility (CV %)			

	0.1 μL	0.5 μL	1 μL	5 μL
water	1.62	1.40	0.76	0.40
10% glycerol	1.03	1.80	2.00	0.50
20% glycerol	2.60	2.00	2.30	0.54
30% glycerol	1.90	3.20	2.20	0.59
40% glycerol	2.35	2.00	1.39	1.96
50% glycerol	3.30	3.70	1.97	1.20
